# The inflammation-reducing compatible solute ectoine does not impair the cytotoxic effect of ionizing radiation on head and neck cancer cells

**DOI:** 10.1038/s41598-019-43040-w

**Published:** 2019-04-29

**Authors:** Thorsten Rieckmann, Fruzsina Gatzemeier, Sabrina Christiansen, Kai Rothkamm, Adrian Münscher

**Affiliations:** 1 Laboratory of Radiobiology & Experimental Radiation Oncology, University Medical Center Hamburg Eppendorf, Hamburg, Germany; 2 Department of Otorhinolaryngology and Head and Neck Surgery, University Medical Center Hamburg Eppendorf, Hamburg, Germany

**Keywords:** Radiotherapy, Head and neck cancer, Oral cancer

## Abstract

Ectoine is a natural protectant expressed by halophile bacteria to resist challenges of their natural environments, such as drought, heat or high salt concentrations. As a compatible solute, ectoine does not interfere with the cell’s metabolism even at high molar concentrations. External application of ectoine results in surface hydration and membrane stabilization. It can reduce inflammation processes and was recently tested in a pilot study for the prevention and treatment of chemotherapy-induced oral mucositis. Oral mucositis is especially frequent and severe in patients with head and neck squamous cell carcinoma (HNSCC), who receive radiotherapy or chemoradiation. It is extremely painful, can limit nutritional intake and may necessitate treatment interruptions, which can critically compromise outcome. As it was recently reported that *in vitro* ectoine has the ability to protect DNA against ionizing irradiation, it was the aim of this study to test whether ectoine may protect HNSCC cells from radiotherapy. Using HNSCC cell lines and primary human fibroblasts, we can show that in living cells ectoine does not impair DNA damage induction and cytotoxicity through ionizing radiation. We therefore conclude that testing the ectopic application of ectoine for its ability to alleviate early radiotherapy/chemoradiation-induced side effects is safe and feasible.

## Introduction

Oral mucositis is one of the most frequent side effects during radiotherapy or chemoradiation (RT/CRT) for head and neck squamous cell carcinoma (HNSCC) affecting the vast majority of patients receiving treatment with curative intent^1^. While oral mucositis as an early event of highly proliferative tissue is in principle reversible due to the regenerative potential of the oral and pharyngeal mucosa, it can be very painful, limit swallowing and adequate nutritional intake and may necessitate the use of a feeding tube or a break in the radiation schedule. The latter can seriously impair treatment efficacy because tumor cells can proliferate during the treatment break and partly repopulate the tumor^2^. The primary cause for radiation-induced mucositis is the killing of epithelial cells, especially the stem cells of the basal cell layer, resulting in mucosal hypoplasia and eventually ulceration. However, also other (sub)mucosal components, such as endothelial cells and fibroblasts are affected. Radiation further induces inflammation triggered in part by oxidative stress and NF–kB-mediated release of inflammatory cytokines, such as interleukin 6 (IL-6), IL-1β and tumor necrosis factor alpha, which again induce NF–kB, resulting in signal amplification^3–5^. Other frequent secondary events include local and systemic bacterial and fungal infections due to open sores in the mucosa. In addition, a reduction in salivary flow can occur towards the end of and beyond treatment, which can further promote such secondary events. Although a large number of oral mucositis treatment protocols exist, there is currently a lack of evidence-based recommendations^6,7^. Many forms of treatment, such as mouthwashes, have been tested in the past. Recently ectoine mouthwash was tested in a pilot study for its effectiveness in the treatment of chemotherapy-induced oral mucositis^8^.

Ectoine is a so called compatible solute expressed by halophile bacteria to protect themselves against stresses related to hostile environments such as osmotic stress, drought or heat through mechanisms including protein stabilization and preservation of turgor presure^9^. Compatible solutes can be expressed at high, molar concentrations without disturbing the metabolic pathways of the producing cell^10^. The principle protective mode of action of the zwitterionic ectoine against the aforementioned denaturing stresses is based on its special physical interaction with water instead of directly interacting with macromolecules^11,12^. According to the “preferential exclusion model” ectoine is excluded from the surface of proteins and their first hydration shell but stabilizes proteins and cell membranes by altering the strength of the hydrogen bonds between ectoine and water molecules in the vicinity^13^. Applied externally, ectoine leads to surface hydration and membrane stabilization of the tissue treated^14^. Based on these effects, ectoine has the potential to reduce inflammation and therefore has been and is further being tested for the treatment of several inflammatory conditions such as allergic rhinitis, atopic dermatitis, chronic obstructive pulmonary disease or acute pharyngitis/laryngitis^15–19^. It is routinely used in many externally applied products, such as skin cream, nasal spray, inhalation solution or eye drops. Recently it was reported that ectoine can induce structural changes in DNA *in vitro*^20^ and even has the potential to protect DNA against ionizing radiation (IR)^21,22^. Three mechanisms have been suggested of how ectoine may protect DNA from the indirect effect of IR, which is caused by ionization of water into free radicals: 1. displacement of water molecules in the vicinity of the DNA, 2. increased inelastic scattering and 3. radical scavenging. To what extent these mechanisms are responsible for the protective effect is currently unclear^21^. These observations of DNA protection were made *in vitro* on purified plasmid DNA outside living cells and the *in vivo* relevance is so far unclear. Given this protective effect it is important to investigate whether ectoine may interfere with the primary mechanism of RT, i.e. induction of lethal DNA damage in tumor cells, when applying ectoine mouthwash in the case of tumors located at or slightly beneath the surface of the oral cavity or pharynx. Therefore, in order to assess the safety and feasibility of testing the external application of ectoine as a protectant against radiation-induced oral mucositis during RT of HNSCC, it was the aim of this study to investigate whether externally applied ectoine can protect HNSCC tumor cells from ionizing radiation.

## Results

In order to evaluate the effect of ectoine on the radiation sensitivity of head and neck cancer cells, we compared the radiation responses of a human papillomavirus (HPV)-negative HNSCC cell line (HSC4), an HPV-positive HNSCC cell line (UD-SCC-2) and primary, normal human fibroblasts (F184; NHF) as an example of normal tissue cells in the presence or absence of ectoine. We used ectoine concentrations ranging from 70 mM to 280 mM, which is the range of concentrations typically used in mouthwash or other medical formulations (usually 1% to 2% (w/w) ectoine corresponding to 70 mM to 140 mM).

### Impact of ectoine on proliferation and colony formation

**Figure 1:**
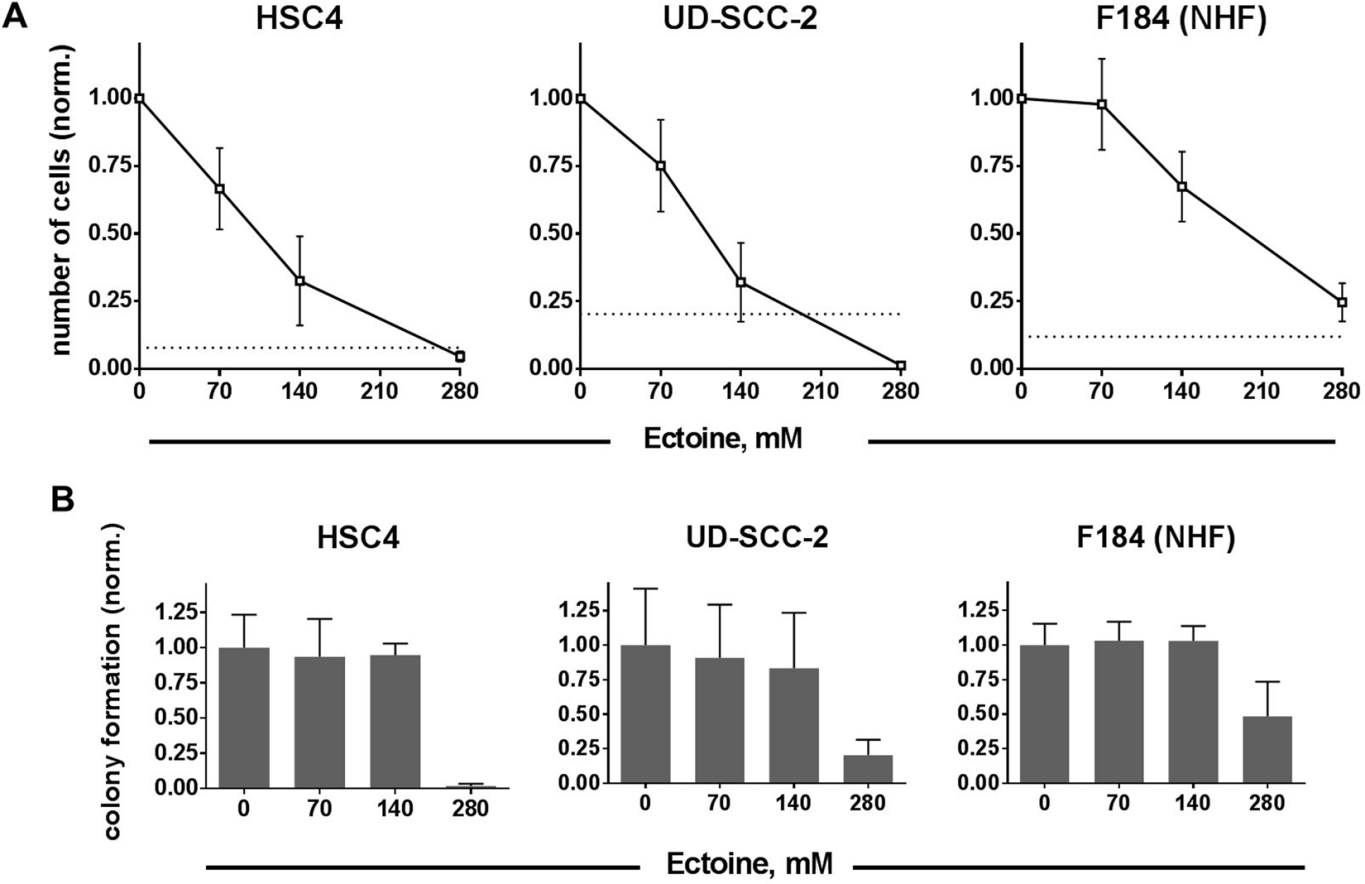
Impact of ectoine on the proliferation and colony formation of HNSCC cells and primary fibroblasts. (**A**) Proliferation. Cells were seeded in defined numbers and treated with the indicated doses of ectoine 24 h later. After further 72 h the resulting numbers of cells were assessed. Values are normalized to the untreated control, dashed lines indicate the numbers of cells initially seeded. (**B**) Colony formation. Exponentially growing cells at approximately 50% density were treated with the indicated concentrations of ectoine for 26 h. Afterwards the cells were seeded in defined low numbers without ectoine to allow colony formation.

For all cell types used, we observed an inhibition of cell proliferation when incubated with high concentrations of ectoine for three days (Fig. 1A). HPV-positive UD-SCC-2 cells demonstrated the strongest response, with virtually all cells being lost at 280 mM ectoine and an almost complete block of proliferation at 140 mM. In contrast, F184 primary fibroblasts did not demonstrate a net cell loss at the concentrations used and showed no or only a moderate inhibition of cell proliferation at 70 and 140 mM ectoine, respectively. As ectoine represents an effective osmolyte, the dose dependent decrease in cell proliferation up to a net cell kill in the tumor cells may be caused by osmotic stress. In fact, challenging the cell lines with osmotic stress through the addition of increasing concentrations of sodium chloride (NaCl) yielded very similar results. We observed a dose dependent decrease in proliferation in all strains with the normal fibroblasts remaining the only strain without a net cell loss at the highest concentration of 154 mM (Supplementary Fig. 1).

Colony formation assays offer a more stringent and robust readout for cytotoxicity than proliferation assays under continuous drug incubation. Using 70 and 140 mM ectoine treatment for 26 h, none of the three cell lines demonstrated an inhibition in the ability to form colonies (Fig. 1B), indicating that the inhibition of proliferation at these concentrations is not caused by a specific cytotoxic effect. At the highest concentration of 280 mM, ectoine induced a reduction of colony formation in all three cell lines ranging from an almost complete suppression in HSC4 cells to about 50% suppression in the normal human fibroblasts. Due to this profound effect and because 280 mM is double the concentration ectoine is typically used as a transiently applied mouthwash most of the following experiments were restricted to 70 and 140 mM ectoine.

### Impact of ectoine on radiation-induced DNA damage

**Figure 2:**
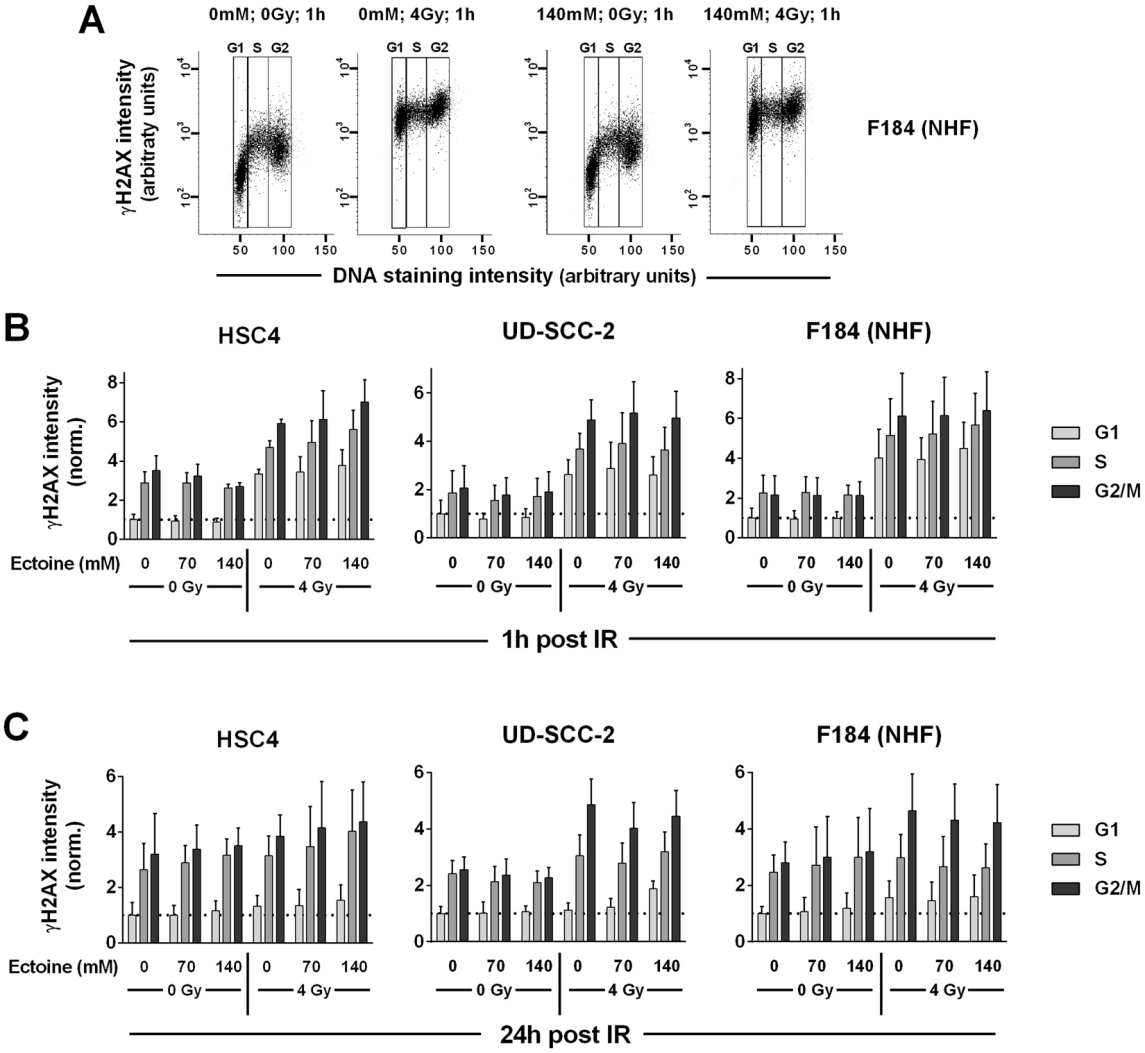
Radiation induced DNA damage. Cells were incubated with the indicated doses of ectoine for 2 h before irradiation with 0 or 4 Gy. After 1 h (induction) or 24 h (residual damage) the cells were harvested, fixed, stained for γH2AX and counterstained for DNA content. (**A**) Exemplary flow cytometric assessment of the γH2AX level in relation to the DNA content in F184 normal human fibroblasts. (**B**) DNA damage induction and (**C**) residual DNA damage levels: Graphs represent the fold change of γH2AX staining intensity of the cell fractions defined by DNA content normalized to the respective untreated G1-fractions.

To assess a possible impact of ectoine on the levels of radiation-induced DNA damage, we quantified the DNA double-strand break marker gamma histone H2AX using flow cytometry. In the vicinity of DNA double-strand breaks or under conditions of replication stalling the histone H2AX is rapidly phosphorylated on S139 by the PI3K-like kinases of the DNA damage response system^23^. This phosphorylated form of H2AX is termed γH2AX. We assessed the induction and residual levels of DNA double-strand breaks by measuring the cellular γH2AX levels at 1 h and 24 h after 4 Gy X-irradiation using flow cytometry. As baseline and radiation-induced yields of γH2AX vary with cell cycle phase, we assessed the γH2AX levels according to the respective DNA content (Fig. 2A). Ectoine treatment alone did not affect baseline cellular γH2AX levels (Fig. 2B). Further, we could not detect any protective impact of ectoine added 2 h prior to X-irradiation on γH2AX induction at 1 h post irradiation. Comparing the γH2AX levels of the three cell lines at 24 h post irradiation UD-SCC-2 showed a big difference between irradiated and non-irradiated S- and G2-phase cells but not in G1, in line with previous data describing a defect in double-strand break repair by homologous recombination in HPV-positive HNSCC^24^ (Fig. 2C). The addition of ectoine did not result in any significant changes of the residual γH2AX levels in the three cell lines.

**Figure 3:**
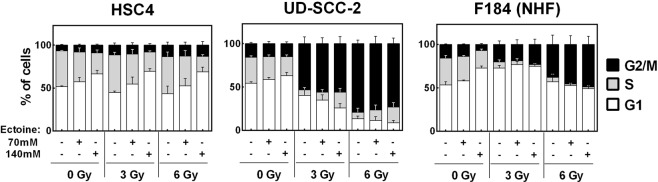
Impact of ectoine on cell cycle distribution. Exponentially growing cells were incubated with the indicated concentrations of ectoine for 2 h before irradiation with the indicated doses and further incubation for 24 h. Afterwards the cells were harvested, fixed and the cell cycle distribution was assessed using propidium iodide staining.

We further assessed the cell cycle distribution of the three cell lines and a possible effect of ectoine treatment (Fig. 3). Sole addition of ectoine enhanced the fraction of cells in the G1-phase, in line with the inhibition of proliferation described above. However these small effects cannot explain the partly profound proliferation inhibition in HSC4 and UD-SCC-2 cells (Fig. 1A). In the case of HPV-negative HSC4 cells, irradiation of up to 6 Gy had no impact on the cell cycle distribution after 24 h regardless of ectoine treatment. As observed previously^25,26^, irradiation induced a profound cell cycle arrest in the G2-phase in HPV-positive UD-SCC-2 cells and in both the G1 and G2 phases in p53-proficient primary fibroblasts (Fig. 3). Here ectoine treatment slightly reduced the fraction of G1-phase cells in irradiated primary fibroblasts and UD-SCC-2, for the latter alongside an increase of S-phase cells. This may be explained by a slightly enhanced G2 arrest (F184) and a reduction in proliferation speed, so that some cells do not reach G2 in this time frame (UD-SCC-2).

### Impact of ectoine on cell survival after radiation

**Figure 4:**
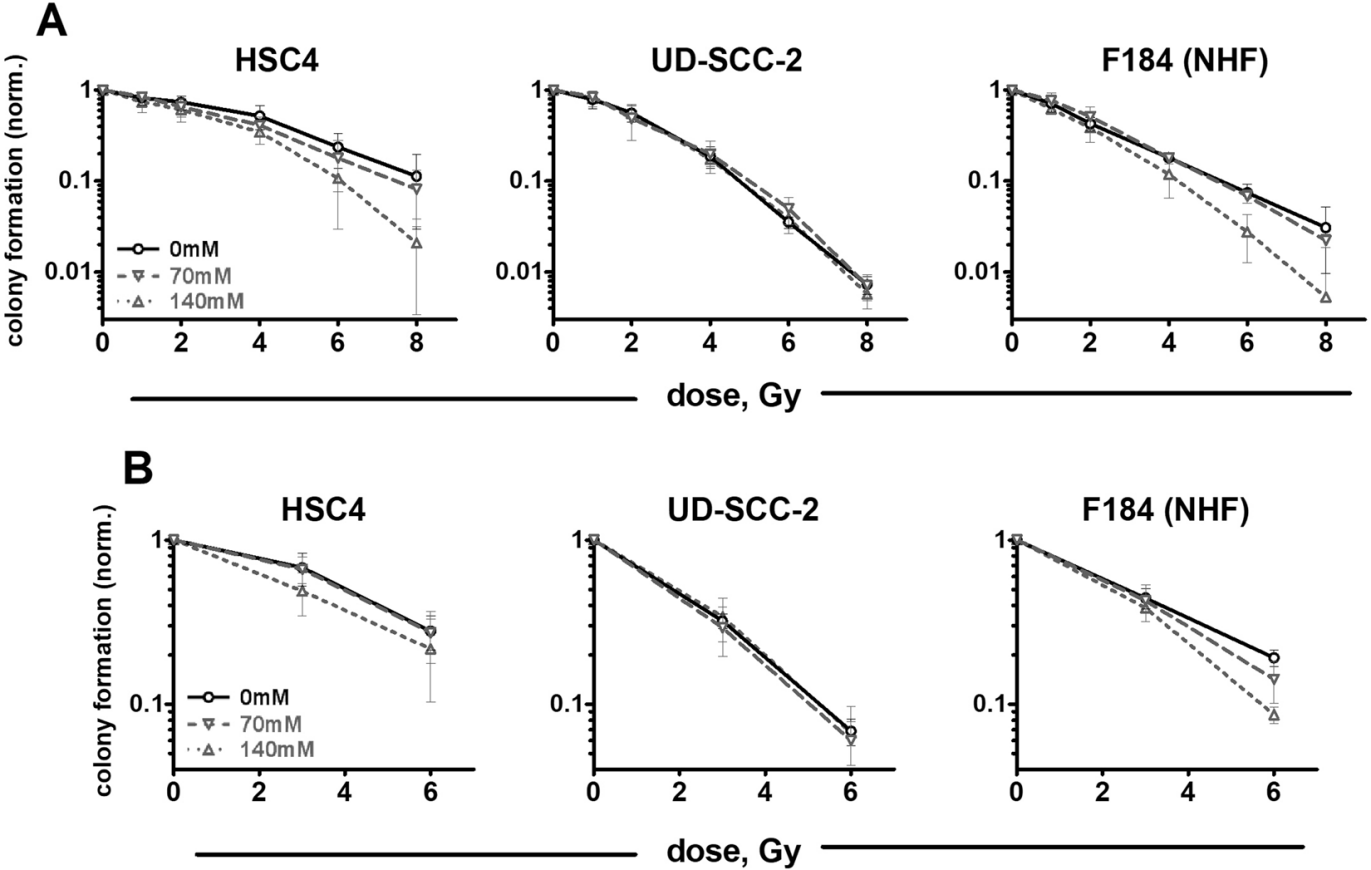
Lack of radioprotection by ectoine. (**A**) Pre-plating: Exponentially growing cells were seeded in defined low numbers and incubated for 24 h. Cells were then treated with the indicated concentrations of ectoine for 2 h before irradiation. 24 h after irradiation the medium was exchanged and cells were incubated until the formation of colonies without ectoine. (**B**) Delayed plating: Exponentially growing cells at approximately 50% confluence were treated with the indicated concentrations of ectoine for 2 h before irradiation. 24 h after irradiation the cells were seeded in defined low numbers without ectoine until the formation of colonies. Lines are shown to guide the eyes.

Colony formation assays after ionizing radiation provide the most robust readout for radiation sensitivity *in vitro*. We performed colony formation assays following a pre-plating approach that allows a higher number of samples as well as a delayed plating approach, where the cells are irradiated under the most physiological conditions at normal density (Fig. 4A,B). Comparing the three cell lines we observed the highest radiation sensitivity in HPV-positive UD-SCC-2 cells and the most pronounced radiation resistance in HPV-negative HSC4. This is in line with previous data^26^ and with the results on residual DNA damage and cell cycle responses described above. Ectoine treatment did not exert a protective effect in any of the cell lines. Instead, treatment with 140 mM ectoine resulted in statistically significant radiosensitization in HSC4 and a trend towards radiosensitization in F184 primary fibroblasts (HSC4: p = 0.0373 and p = 0.0224 and F184: p = 0.0528 and p = 0.0576 for the respective pre-plating and delayed plating experiments). Treatment with the toxic concentration of 280 mM ectoine resulted in a severe radiosensitization of the F184 cells (p < 0.001) confirming the trend seen with 140 mM (Supplementary Fig. 2). Most importantly, treatment with 70 mM ectoine which represents the most relevant concentration when considering the usage of ectoine as mouthwash did not significantly impact on radiosensitivity in any of the cell lines tested (Fig. 4).

## Discussion

The main goal of this study was to test whether the external application of the compatible solute ectoine may have a protective effect on HNSCC tumor cells when treated with radiotherapy. Since it was recently reported that ectoine can in principle protect DNA against ionizing radiation^21,22^ this question needed to be addressed before ectoine can be tested in the clinic for its potential to alleviate radiation-induced oral mucositis. We applied 70, 140 and 280 mM ectoine which corresponds to the half, full and double concentration of the 2% ectoine typically used for external application. To use stringent conditions, we added ectoine for 2 h prior to irradiation and kept the compound on the cells afterwards for at least 24 h, which clearly represents overtreatment compared to the transient, short-term use as a mouthwash. We did not observe any changes in radiation-induced DNA double-strand break formation (Fig. 2) or a radioprotective effect in colony formation assays, neither in the HNSCC cell lines nor in primary fibroblasts (Fig. 4). These results therefore suggest that externally applied ectoine does not confer radioprotection by interfering with the primary effects of radiation, namely the induction of lethal DNA damage. A simple explanation would be that, since the zwitterionic ectoine cannot pass cellular membranes, it is not present inside the nuclei of the treated cells in high enough concentrations to have any effect on the DNA damage induction through ionizing radiation. Buommino *et al*. reported that the ectoine concentration in the supernatant of human keratinocyte cultures decreases during a 24 h incubation period, with no further decrease being observed after 48 h of incubation. They speculated that this decrease may be caused by intracellular accumulation, adhesion on cell membranes or metabolization^27^. To our knowledge, no further data have been reported so far that would specifically demonstrate a possible accumulation of ectoine inside mammalian cells or nuclei, and the concentrations used to induce protection of plasmid DNA from ionizing radiation in cell free assays are actually quite high, ranging from 200 mM to 1 M^21,22^, with a minimal concentration yet to be defined.

Also in line with the lack of radioprotection reported here are recent data from extremophile bacteria. These data show that there is no correlation between desiccation tolerance, which involves the expression of a variety of compatible solutes, and the tolerance towards ionizing radiation^28^. In a comparison of five thermophilic bacteria and archaea one of the strains showed an enhanced resistance against ionizing radiation when cultured under conditions stimulating compatible solute induction. According to those data, radioprotection by the endogenous expression of compatible solutes is possible but appears to be a rather rare event, likely dependent on the individual mixture of compatible solutes of a given strain^29^. Comparing different specific compatible solutes, the external addition of 500 mM ectoine resulted in the most effective protection against desiccation, but did not result in radiation protection^29^. It has to be noted, however, that irradiation of the ectoine-treated cells was performed with UV-C where DNA damage formation is far less dependent on the radiolysis of water molecules and a different spectrum of damages with far fewer double-strand breaks is induced. Regarding the mechanisms of protection against desiccation and the lack of radioprotection, also for these bacterial strains the authors state that it has not been completely clarified whether externally applied ectoine is incorporated into the cells or acts from the outside^29^, although here an uptake appears likely, taking into account the effective protection against desiccation-derived osmotic stress.

The dose dependent decrease in cell proliferation and the profound cell kill at 280 mM observed here (Fig. 1) may be explained through different mechanisms. One such mechanism could be the inhibition of protein function through ectoine, which has been demonstrated for a small number of proteins, e.g. bacterial restriction endonucleases^30,31^. Meyer *et al*. have reported of DNA single-strand break induction through ectoine in aqueous solution but this effect occurred only at for mammalian cells unphysiologically low pH and high concentrations (500 mM but not 100 mM ectoine)^20^. As ectoine represents an effective osmolyte a more likely explanation could be osmotic stress, which in this case both tumor cell lines would be less capable of handling compared to the primary fibroblasts. In fact, the decrease in proliferation under ectoine treatment was quite similar to the decrease upon addition of NaCl at comparable increases in osmolarity (Supplementary Fig. 1). Since colony formation after a 26 h incubation period with 140 mM ectoine was barely affected (Fig. 1B), a specific cytotoxic effect of the compound seems rather unlikely. Osmotic stress may also be the reason for the somewhat unexpected radiosensitization observed with 140 and 280 mM ectoine in HSC4 and primary fibroblasts (Fig. 4, Supplementary Fig. 2). In mammalian cells osmotic stress through NaCl was shown to induce a substantial amount of special double-strand breaks in genomic regions devoid of genes, so called *genomic deserts*^32^. Interestingly these breaks are not decorated by γH2AX. Through interaction with radiation-induced DSBs followed by misrepair and lethal chromosomal aberrations such extra-lesions could well explain the radiosensitization observed. However, whether this phenomenon also occurs upon osmotic stress through ectoine has never been investigated. With regard to our main goal of risk assessment of ectoine-mouthwash, where the ectoine concentration will be rapidly reduced by dilution through saliva and subsequent swallowing, a continuous 26 h treatment represents a harsh condition chosen to exclude tumor cell radioprotection and we do not believe that a radiosensitizing effect on neither tumor nor normal tissue cells can be extrapolated from the 140 mM dose response curves.

RT/CRT induced oral mucositis is a multistep process involving initiation through mucosal cell killing, inflammation through immune signalling and signal amplification, ulceration, infections and finally healing and restoration^5^. This provides opportunities for various potential interventions such as radical scavenging, reduction of immune stimulation or accelerated healing. Recently, promising results have been reported using the superoxide dismutase mimetic GC4419^33^ for which breakthrough therapy designation was granted by the FDA and a phase 3 clinical was recently started (ClinicalTrials.gov: NCT03689712). However, it is likely that the most effective treatment and prevention of this multistep process will be achieved through an optimal combination of different substances and mechanisms. Here, ectoine may, due to its well-described inflammation-reducing, moisturizing as well as membrane- and cell-protective properties, help to achieve best results in the reduction of inflammation, ulceration and secondary infections. In this regard we would generally recommend to test a possible benefit of ectoine when added to and not in comparison to current best care. Against this background our data clearly demonstrate that the application of ectoine does not interfere with the primary cytotoxic effects of radiation on HNSCC tumor cells, despite the recently described protection of cell free DNA from ionizing irradiation in aqueous solution.

## Methods

### Cells and cell culture

All cell lines were grown in DMEM (Gibco) supplemented with 10% fetal bovine serum (Biochrom AG) at 37 °C, 5% CO2 and 100% humidification. HPV-negative HNSCC cells HSC4, HPV-positive HNSCC cells UD-SCC-2 and primary human fibroblasts F184 were described previously^25,26^. Ectoine (purity >99%) was a kind gift of A. Bilstein of bitop AG.

### Cell proliferation

For cell proliferation analysis, cells were seeded in defined numbers into T25 cell culture flasks. The medium was changed to ectoine-containing medium 24 hours later. The numbers of resulting cells were assessed after 3 days of incubation with ectoine using a Coulter Counter (Beckmann).

### Assessment of DNA damage

The induction as well as the residual levels of radiation-induced DNA damage were assessed through γH2AX quantification by flow cytometry. Cells were harvested, fixed with PBS/4% formaldehyde for 10 min and permeabilized with PBS/0.2% Triton X-100 before blocking overnight with PBS/1% BSA/0.2% Triton X-100. The cells were subsequently incubated (1 h; room temperature) with a mouse-anti- γH2AX antibody (clone JBW301, Millipore) in blocking solution, washed three times with PBS/0.1% Tween20 before incubation (1 h; room temperature) with anti-mouse DyLight488 (Jackson Immunoresearch) and were then washed again three times. DNA counterstaining was performed using FxCycle FarRed (Molecular Probes) plus 300 ng/ml RNAse A and 0.2% Triton X-100 for 30 min at room temperature in the dark. Flow cytometric analysis was performed using a FACS Canto with FACS Diva Software (Becton Dickinson).

### Cell cycle assessment

Cells were harvested, fixed with 70% ethanol, briefly washed with PBS/0.2% Triton X-100 and subsequently incubated with 300 ng/ml RNAse A and 10 µg/ml propidium iodide in PBS plus 0.2% Triton X-100 for 30 min at room temperature in the dark. Flow cytometric analysis was performed using a FACS Canto with FACS Diva Software (Becton Dickinson). The fraction of cells in the respective cell cycle phases was calculated using ModFit LT^TM^ software (Verity Software House, Inc.).

### Colony formation assay

Cell survival after ionizing radiation was assessed in pre-plating as well as delayed plating colony formation assay. Pre-plating: Exponentially growing cells were seeded in defined numbers into 6well plates. Ectoine was added to the cultures after 24 hours. Two hours later the cells were irradiated and incubated for 24 hours before media exchange and further incubation until the formation of colonies without ectoine. Delayed plating: Exponentially growing cells were treated with ectoine and irradiated after 2 hours of incubation. 24 h hours post irradiation the cells were seeded in defined numbers into T25 cell culture flasks without the addition of ectoine and incubated until the formation of colonies. For radiosensitive UD-SCC-2 cells irradiated samples were allowed to grow for an extended period of time, as colony formation was apparently delayed. The number of colonies containing more than 50 cells was assessed. In the case of F184 the medium was changed to a 1/1 mixture of DMEM plus 10% FBS and Amniomax C-100 medium plus 7,5% Amniomax Supplement (both Gibco) and 7,5% FBS 1 week after seeding to facilitate colony formation. Single experiments always contained the full set of ectoine concentrations and radiation doses.

### X-irradiation

Cells were irradiated at room temperature with 200 kV X-rays using a Gulmay RS225 X-ray source (Gulmay Medical Ltd) at 200 kV, 15 mA with 0.8 mm Be + 0.5 mm Cu filtering. The dose rate was set to 1.2 Gy/min by positioning the sample in the correct distance from the X-ray source. The dose rate was tested routinely once every two weeks using a PTW-SN 4 ionization chamber (PTW Freiburg GmbH) and annually using Gafchromic EBT3 film, with spatial and temporal dose variations below 3%.

### Data evaluation

Data analysis was performed using Excel (Microsoft) and GraphPad Prism 6 (GraphPad Software). Statistical evaluation of colony formation after radiation was performed using a repeated measures two-way ANOVA test. All experiments were performed at least three times and values presented are means ± SD.

## Supplementary information

Dataset 1
