# Supplementary material for: The inflammation-reducing compatible solute ectoine does not impair the cytotoxic effect of ionizing radiation on head and neck cancer cells
Source: Sci Rep. 2019 Apr 29;9:6594. doi: 10.1038/s41598-019-43040-w (PMC6488604; doi:10.1038/s41598-019-43040-w)
Supplement: Supplementary file 1 — Dataset 1 [file 41598_2019_43040_MOESM1_ESM.pdf]

Supplementary information

The inflammation-reducing compatible solute ectoine does not impair the cytotoxic effect of ionizing radiation on head and neck cancer cells

T. Rieckmann, F. Gatzemeier, S. Christiansen, K. Rothkamm*, A. Münscher*

* equal contribution

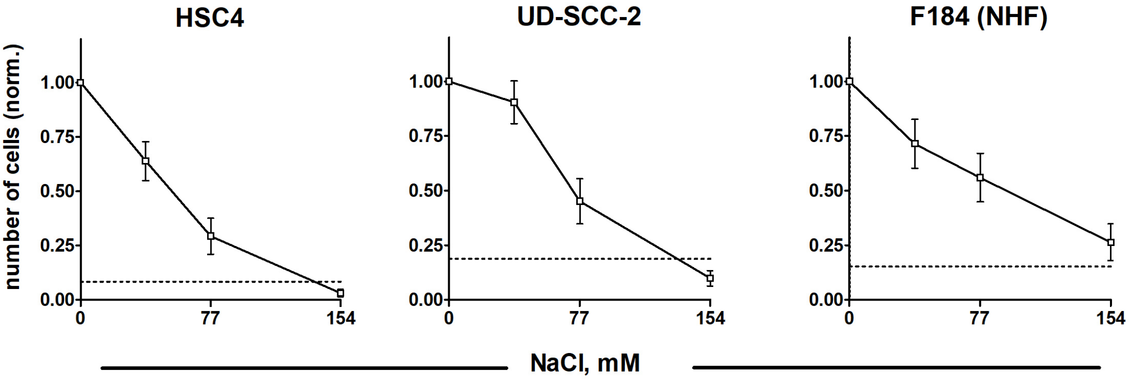

Supplementary Figure 1. Impact of osmotic stress through NaCl on the proliferation of HNSCC cells and primary fibroblasts. Cells were seeded in defined numbers in cell culture media. After 24h the cultures we supplemented with NaCl increasing the total NaCl concentration by the indicated doses. After further 72 h the resulting numbers of cells were assessed. Values are normalized to the untreated control, dashed lines indicate the numbers of cells initially seeded. 154 mM NaCl refers to the 0.9 % NaCl of standard isotonic saline solutions. The increase in osmolarity through the addition of 154 mM NaCl is comparable to the increase through 280 mM ectoine (both approximately 300 mosmol/l, doubling normal osmolarity).

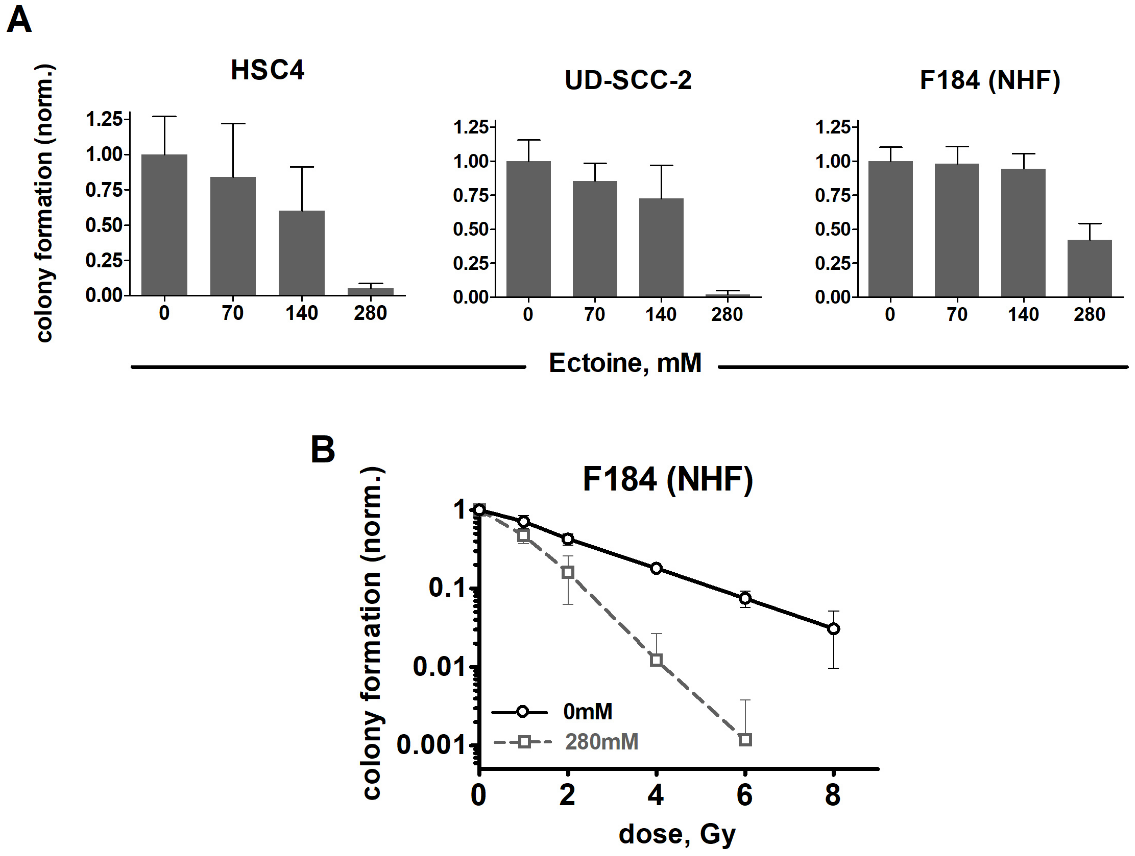

Supplementary Figure 2. Impact of ectoine on the colony formation of HNSCC cells and primary fibroblasts under pre-plating conditions. A) Colony formation without irradiation. Exponentially growing cells were seeded in defined low numbers and incubated for 24 h. Cells were then treated with the indicated concentrations of ectoine for 26 h. Afterwards the medium was exchanged and cells were incubated until the formation of colonies without ectoine. The stronger inhibition of colony formation in UD-SCC-2 as compared to delayed plating (Figure 1B) may be explained by additional inhibitory effects of ectoine on cell adhesion at low cell density. B) Radiosensitization through high dose ectoine. Exponentially growing F184 normal fibroblasts were seeded in defined low numbers and incubated for 24 h. Cells were then treated with the indicated concentrations of ectoine for 2 h before irradiation. 24 h after irradiation the medium was exchanged and cells were incubated until the formation of colonies without ectoine. Treatment with ectoine induced statistically significant radiosensitization ($p=0.0005$; repeated measures two-way ANOVA test). Lines are shown to guide the eyes. Note that the lower part of the error bars of the 4 and 6 Gy dose points of 280 mM treatment could not be plotted because they extend to negative values.
